# Impact of SARS-CoV-2 Infection on the Course of Inflammatory Bowel Disease in Patients Treated with Biological Therapeutic Agents: A Case-Control Study

**DOI:** 10.3390/biomedicines10040843

**Published:** 2022-04-03

**Authors:** Alfredo Papa, Franco Scaldaferri, Marcello Covino, Antonio Tursi, Federica Furfaro, Giammarco Mocci, Loris Riccardo Lopetuso, Giovanni Maconi, Stefano Bibbò, Marcello Fiorani, Lucrezia Laterza, Irene Mignini, Daniele Napolitano, Laura Parisio, Marco Pizzoferrato, Giuseppe Privitera, Daniela Pugliese, Tommaso Schepis, Elisa Schiavoni, Carlo Romano Settanni, Lorenzo Maria Vetrone, Alessandro Armuzzi, Silvio Danese, Antonio Gasbarrini

**Affiliations:** 1 Gastroenterology Department, CEMAD, Center for Diagnosis and Treatment of Digestive Diseases, Fondazione Policlinico Gemelli, IRCCS, 00168 Roma, Italy; franco.scaldaferri@policlinicogemelli.it (F.S.); lorislopetuso@libero.it (L.R.L.); stefano.bibbo@policlinicogemelli.it (S.B.); marcellofiorani94@gmail.com (M.F.); lucrezia.laterza@policlinicogemelli.it (L.L.); irene.mignini@gmail.com (I.M.); daniele.napolitano@policlinicogemelli.it (D.N.); lparisio11@gmail.com (L.P.); marco.pizzoferrato1@policlinicogemelli.it (M.P.); gpp.privitera@icloud.com (G.P.); daniela.pugliese@policlinicogemelli.it (D.P.); tommaso.schepis@gmail.com (T.S.); elisa.schiavoni@policlinicogemelli.it (E.S.); carloromano.settanni@policlinicogemelli.it (C.R.S.); vetrone.md@gmail.com (L.M.V.); alessandro.armuzzi@policlinicogemelli.it (A.A.); antonio.gasbarrini@policlinicogemelli.it (A.G.); 2 Department of Translational Medicine and Surgery, School of Medicine, Università Cattolica del S. Cuore, 00168 Roma, Italy; macovino@gmail.com; 3 Emergency Department, Fondazione Policlinico Gemelli, IRCCS, 00168 Roma, Italy; 4 Territorial Gastroenterology Service, ASL BAT, 70031 Andria, Italy; antotursi@tiscali.it; 5 IBD Center, Department of Gastroenterology, Humanitas Clinical and Research Center—IRCCS, 20089 Rozzano, Italy; federica.furfaro28@gmail.com; 6 Gastroenterology Department, “Brotzu” Hospital, 09121 Cagliari, Italy; giammarco.mocci@gmail.com; 7 Department of Medicine and Ageing Sciences, “G. d’Annunzio” University of Chieti-Pescara, 66100 Chieti, Italy; 8 Center for Advanced Studies and Technology (CAST), “G. d’Annunzio” University of Chieti-Pescara, 66100 Chieti, Italy; 9 Gastroenterology Department, “L. Sacco” University Hospital, 20157 Milan, Italy; giovanni.maconi@unimi.it; 10 Gastroenterology and Endoscopy Unit, IRCCS Ospedale San Raffaele, University Vita-Salute San Raffaele, 20132 Milan, Italy; sdanese@hotmail.com

**Keywords:** SARS-CoV-2, inflammatory bowel disease, biological agents

## Abstract

Severe acute respiratory syndrome coronavirus 2 (SARS-CoV-2) infection has raised concerns in patients with inflammatory bowel disease (IBD), not only due to consequences of coronavirus disease 2019 itself but also as a possible cause of IBD relapse. The main objective of this study was to assess the role of SARS-CoV-2 in IBD clinical recurrence in a cohort of patients undergoing biological therapy. Second, we evaluated the difference in C-reactive protein (CRP) levels between the start and end of the follow-up period (ΔCRP) and the rate of biological therapy discontinuation. Patients with IBD positive for SARS-CoV-2 infection were compared with non-infected patients. IBD recurrence was defined as the need for intensification of current therapy. We enrolled 95 IBD patients with SARS-CoV-2 infection and 190 non-infected patients. During follow-up, 11 of 95 (11.6%) SARS-CoV-2-infected patients experienced disease recurrence compared to 21 of 190 (11.3%) in the control group (*p* = 0.894). Forty-six (48.4%) SARS-CoV-2-infected patients discontinued biological therapy versus seven (3.7%) in the control group (*p* < 0.01). In the multivariate analysis, biological agent discontinuation (*p* = 0.033) and ΔCRP (*p* = 0.017), but not SARS-CoV-2 infection (*p* = 0.298), were associated with IBD recurrence. SARS-CoV-2 infection was not associated with increased IBD recurrence rates in this cohort of patients treated with biological agents.

## 1. Introduction

Coronavirus disease 2019 (COVID-19), which is caused by severe acute respiratory syndrome coronavirus 2 (SARS-CoV-2), emerged in early 2020 as a global public health crisis [1]. By the end of January 2022, 380 million cases of COVID-19 and over 5.6 million deaths had been reported worldwide since the start of the pandemic [2]. The individuals most at risk of severe outcomes, defined by intensive care unit (ICU) admission or death, are the elderly, as well as frail individuals with multiple morbidities such as neoplastic, degenerative, and chronic diseases [3,4]. The latter group includes patients with immune-mediated inflammatory diseases such as inflammatory bowel disease (IBD), particularly those undergoing treatment with immunosuppressive or biological agents [5,6]. These drugs potentially predispose patients to an increased risk of infectious complications, including viral infections, by weakening the immune system [7,8]. In addition, although gastrointestinal symptoms are reported to be common clinical manifestations of COVID-19 in patients with IBD [9], limited data are available on the effect of SARS-CoV-2 infection on IBD flare-ups. This is particularly relevant in patients experiencing a more severe clinical course, including those undergoing treatment with biological agents. Therefore, this study aimed to evaluate the impact of SARS-Cov-2 infection on the clinical course of IBD in a cohort of patients receiving treatment with biological agents compared with that in non-infected patients.

## 2. Materials and Methods

### 2.1. Cases and Controls

**Figure 1:**
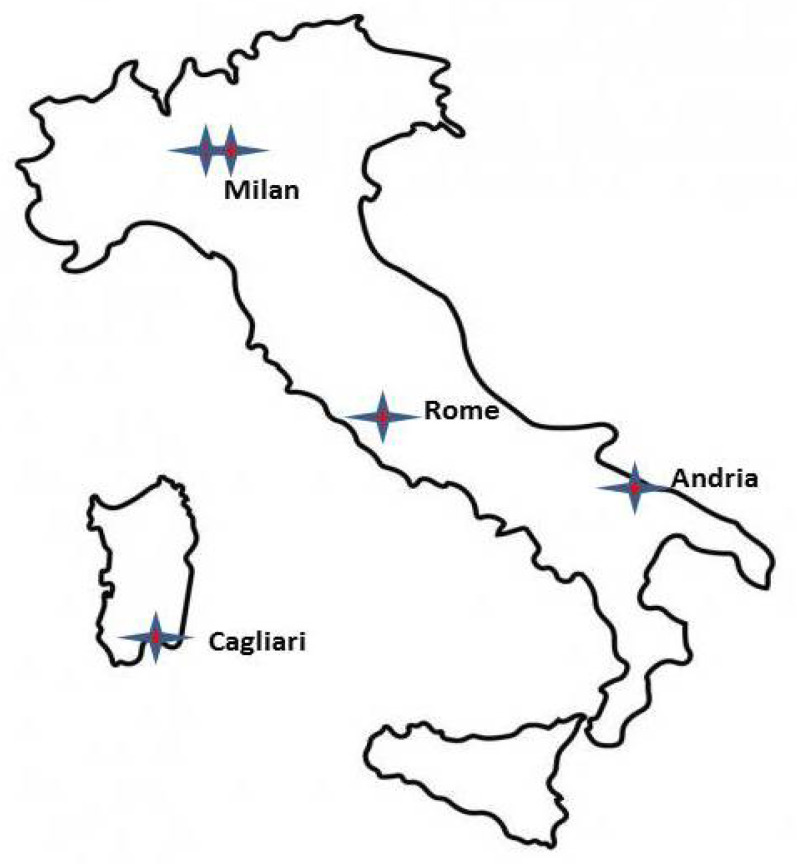
Map of Italy depicting the locations of the centers included in the study: two in the north of Italy (Milan), one in the center (Rome), one in the south (Andria), and one in Sardinia (Cagliari).

We conducted a multicenter study that included patients from five Italian IBD treatment centers (Figure 1). We retrospectively reviewed the medical records of adult patients with IBD (over 18 years of age) who had been diagnosed with Crohn’s disease (CD) or ulcerative colitis (UC) and who were being treated with one of the following biological agents: infliximab (IFX), adalimumab (ADA), vedolizumab (VED), or ustekinumab (UST). From October 2020 to March 2021, all consecutive patients with IBD who were diagnosed with SARS-CoV-2 infection (cases) were included in the study and compared with SARS-Cov-2-negative patients (controls). Only patients with stable, inactive IBD, defined as those who experienced no change in therapy in the previous three months, were included in the study. Pregnant women and patients with incomplete dataset parameters were excluded from the study. Cases were matched to controls at a 1:2 ratio based on disease type (CD or UC) and biological agent type to reduce any potential bias resulting from the influence of confounding variables. Follow-up began on the day of the first SARS-CoV-2-positive oropharyngeal molecular swab for cases and on the same day (±14 days) for the controls. Follow-up ended in cases of IBD reactivation or at the final administration of a biological agent. Demographic and clinical features (according to the Vienna Classification), including the type and dosage of biological agents administered, concomitant medications used, previous IBD-related surgeries, extraintestinal manifestations, and smoking habits were recorded. C-reactive protein (CRP) concentrations (expressed in mg/dL) were recorded at the time of study inclusion and at the end of the follow-up period, and the difference between these two values, defined as ΔCRP, was assessed for each patient. The severity of COVID-19 was classified as follows: asymptomatic; mild (for symptomatic patients not requiring oxygen supplementation or hospitalization); and severe (for symptomatic patients requiring hospitalization, including stays in the ICU or oxygen supplementation).

### 2.2. Study Outcomes

The primary outcome of the study was the IBD recurrence rate, defined as the need for therapeutic intensification, at the discretion of the gastroenterologist, to regain control of disease activity. More specifically, we considered one of the following events as evidence of therapeutic intensification: (1) the optimization of ongoing biological therapy, defined as an increase in dosage or a shortening of the interval between two successive administrations (or both); (2) biological switch, defined as the use of a different biological agent belonging to the same class; (3) biological swap, defined as the use of a biological agent belonging to a different class; (4) the addition of a treatment adjunct to the current biological therapy, including immunosuppressant agents and/or corticosteroids, either systemic or intestinal-releasing (also topical), or mesalamine (also topical); (5) the discontinuation of biological therapy and initiation of tofacitinib treatment (only for patients with UC); and (6) discontinuation of biological therapy and subsequent surgery. The secondary study outcomes involved the assessment of ΔCRP values and the discontinuation rate of biological agents in the cases and controls.

### 2.3. Statistical Methods

Continuous variables are presented as median [interquartile range (IQR)] values and compared between groups using the Mann–Whitney U test. Categorical variables are presented as absolute numbers (percentages) and compared using the chi-square test or Fisher’s exact test, as appropriate. A two-sided *p*-value ≤ 0.05 was regarded as statistically significant. Study variables significantly associated with IBD relapse in the univariate analysis were entered into a multivariate Cox regression model to identify the independent predictors of IBD relapse. SARS-CoV-2 infection was forced into the logistic regression model. Logistic regression results are presented as hazard ratios (HRs) with 95% confidence intervals (CIs). All data were analyzed using SPSS v25^®^ software (IBM, Armonk, NY, USA). The data underlying this article will be shared upon reasonable request to the corresponding author.

### 2.4. Sample Size Calculation

As 17 variables were entered into the logistic regression model, a total of 170 patients with IBD would have been required in the study cohort to ensure satisfactory parameter estimation. The number of patients included in the study cohort largely exceeded these requirements.

## 3. Results

Overall, 285 patients with IBD who were treated with biological agents were enrolled, 95 of whom tested positive for SARS-CoV-2 infection and 190 of whom tested negative. The clinical characteristics of the patients are presented in Table 1. The median follow-up period of the study was 21 (16–26) weeks for patients in the SARS-CoV-2 group and 23 (16–25) weeks in the non-infected control group (*p* = 0.959). During the follow-up period, IBD relapse occurred in 32 patients (11.2%), 12 of whom had UC and 20 of whom had CD. Overall, 11 out of 95 (11.6%) patients with IBD who tested positive for SARS-CoV-2 infection experienced disease relapse compared to 21 out of 190 (11.3%) in the control group, without a significant difference between the two groups (*p* = 0.894).

By dividing the patients according to the different biological agents used (IFX, ADA, VED, and UST), no differences were observed in the disease relapse rate between the different groups in positive and negative COVID-19 patients (*p* = 0.177 and *p* = 0.776, respectively). Although we overall compare patients on anti-TNF-alpha (IFX and ADA) therapy with those on other biological agents (VED and UST), the difference in disease reactivation rate in both COVID-19 positive and COVID-19 negative ones was not statistically significant (*p* = 0.620 and *p* = 0.360, respectively).

**Figure 2:**
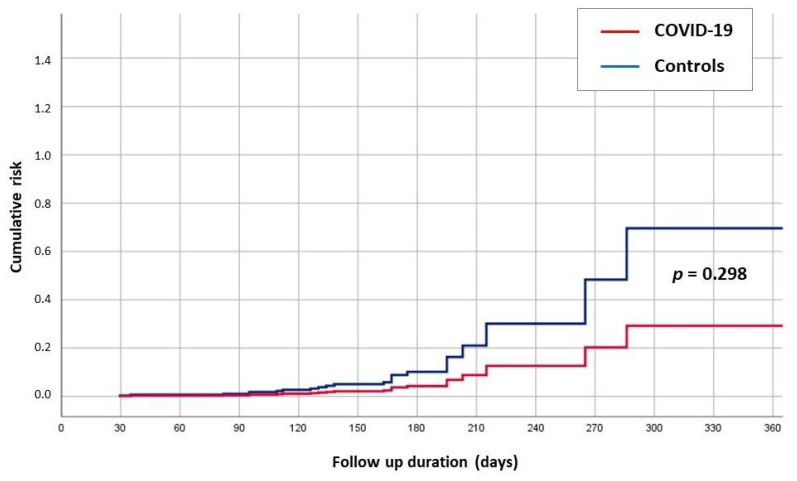
The cumulative risk of IBD recurrence in patients with COVID-19 versus controls (non-infected patients with IBD). IBD, inflammatory bowel disease; COVID-19, coronavirus disease 2019.

Forty-six (48.4%) patients with SARS-CoV-2 infections discontinued biological therapy, compared to just seven (3.7%) patients in the control group (*p* < 0.01). The mean number of non-administered doses of biological agents was significantly higher in the SARS-CoV-2-positive patients than in the controls {2 (1–2) versus 1 (1–1), respectively; *p* = 0.008}. None of the patients in either group discontinued biological therapy because of adverse events or intolerance experienced during the follow-up period. In the univariate analysis, only the discontinuation of biological therapy (*p* < 0.001) and ΔCRP (*p* = 0.002) were associated with IBD flare-ups (Table 2). In the multivariate analysis, both factors, but not SARS-CoV-2 infection (Figure 2), were independently associated with IBD exacerbation (*p* = 0.017 and *p* = 0.033 for ΔCRP and discontinuation of biological agents, respectively) (Table 3). The clinical features of the SARS-CoV-2 patients are reported in Table 4. COVID-19 severity, as previously defined, did not differ between patients with CD and those with UC (*p* = 0.168). Among the patients with COVID-19, the number of discontinued doses of biological agents was significantly greater in those with CD than in those with UC (*p* = 0.003).

## 4. Discussion

It is well known that gastrointestinal symptoms, including nausea, diarrhea, and abdominal pain, are frequently reported among patients with COVID-19, and increased fecal calprotectin levels are also observed in a substantial proportion of patients, thereby confirming digestive tract involvement in COVID-19 [10]. However, in most cases, digestive symptoms disappear when the SARS-CoV-2 infection resolves, despite the fact that some functional symptoms triggered by the virus itself may persist for a variable length of time [11]. To date, the accumulated data have not indicated a more severe course of COVID-19 in patients with IBD than in non-IBD subjects [6]; however, the effect of SARS-CoV-2 infection on the clinical activity of IBD has not yet been fully clarified by studies aiming to investigate that specific relationship. Our data, obtained from a homogeneous and relatively large cohort of patients with IBD who were undergoing treatment with biological agents, showed that SARS-CoV-2 infection was not associated with an increased rate of disease flare-ups compared to rates in non-infected patients. Indeed, in the present study, the relapse rate was 11.6% in SARS-CoV-2-infected patients and 11.3% in SARS-CoV-2-negative controls over a median follow-up period of approximately 5 months. Recently, a Canadian study reported that among 82 patients with IBD and COVID-19, eight (9.5%) experienced IBD disease reactivation [12]. However, in this population, only 72% were being treated with biological agents, 30/59 (50.8%) of whom discontinued therapy after infection [12]. A Danish COVID-IBD cohort study, which included 516 patients with IBD diagnosed with COVID-19, reported additional data related to short-term (within 12 weeks following SARS-CoV-2 infection) and long-term (after 12 weeks) clinical relapse after COVID-19 [13]. Overall, 10 out of 319 (3.1%) patients with UC and 14/197 (7.1%) with CD experienced clinical relapse over a median follow-up period of 5.1 (4.5–7.9) months (13). However, that IBD cohort included only 42/319 (13.2%) patients with UC and 76/197 (38.6%) patients with CD who were being treated with biological agents at the time of COVID-19 diagnosis [13]. Another study from the United States, which included 118 patients with IBD with SARS-CoV-2 infections, did not report any significant consequences on disease activity over a 6-month post-COVID-19 follow-up period, consistent with our own results [14]. Furthermore, their findings did not indicate any significant need for IBD-related surgery or hospitalization after recovery from COVID-19 [14]. Finally, the impact of COVID-19 on the course of IBD was investigated in a large, retrospective cohort study based on a multi-institutional research platform called TriNetX (Cambridge, MA, USA), which included 4310 patients with IBD who had been diagnosed with COVID-19 and 851,163 patients with no history of IBD who had been diagnosed with COVID-19 [15]. Among the patients with IBD who were being actively treated with biological agents at the time of the COVID-19 diagnosis, 8.55% experienced disease exacerbation at 3 months [15]. Furthermore, contrary to our results, the authors of that study reported that, overall, patients with IBD who were diagnosed with COVID-19 had a higher risk of IBD exacerbation at 3 months of follow-up, regardless of treatment type, than controls without COVID-19 (6.77% vs. 5.08%, respectively; *p* < 0.01) [15]. However, this result refers not only to patients being treated with biological agents but also to the entire patient population, and the follow-up time was shorter than that of the present study. In fact, in our study, only patients undergoing stable treatment with biological agents were enrolled because they represented a population of individuals who underwent regular and relatively frequent clinical and laboratory testing, especially during the pandemic period. In addition, such patients experience a more severe disease course and are therefore at greater risk of disease flare-ups than patients undergoing non-biological maintenance therapy.

Other studies have recently investigated the effect of SARS-CoV-2 vaccination on the onset of the clinical recurrence of IBD. In the PREVENT-COVID (Partnership to Report Effectiveness of Vaccination in Populations Excluded from iNitial Trials of COVID) study, only 71/3316 (2.1%) individuals met the criteria for IBD exacerbation after vaccination [16]. After vaccination, IBD flare-ups occurred in 48 of 1908 (2.5%) patients receiving the Pfizer/BioNTech BNT162b2 vaccine, in 22 of 147 (1.8%) patients receiving the Moderna mRNA-1273 vaccine, and in 1 of 161 (0.6%) patients receiving the Janssen Ad26.COV2.S. vaccine [16]. The present study did not include patients who had been vaccinated against SARS-CoV-2, as the vaccines were not yet widely available for patients with IBD in Italy at the time of enrolment. However, the conclusions of our study are equally reliable, even considering that most patients with IBD are currently vaccinated against COVID-19, as this variable does not appear to affect the reported data. Interestingly, we found a significant association between the discontinuation of biological therapy and the clinical recurrence of IBD, regardless of COVID-19 status. Therefore, this finding reinforces the recommendation not to discontinue biological therapy to avoid IBD relapses [17,18]. In our cohort, approximately 48% of the patients with COVID-19 discontinued biological therapy. Data from the Surveillance Epidemiology of Coronavirus under Research Exclusion for Inflammatory Bowel Disease (SECURE-IBD) registry confirmed our findings, as 40% of patients discontinued anti-tumor necrosis factor alpha (TNF-α) therapy after COVID-19 diagnosis and 62% discontinued anti-TNF-α and immunomodulator combination therapy [19]. Therefore, we believe that it is reasonable to temporarily suspend biological therapy only in the case of symptomatic COVID-19 and to resume it immediately after the remission of symptoms without waiting for the disappearance of SARS-CoV-2.

This study has some limitations that should be considered when interpreting the results. First, some data may be missing due to the retrospective nature of the study design, such as endoscopic data, levels of fecal calprotectin, and previous treatment with other biologics. Second, the clinical recurrence of IBD was inferred in each case from the gastroenterologist’s decision to intensify therapy to control disease exacerbation. However, this variable proved reliable, as it was significantly associated with the difference between the CRP values measured at the time of study inclusion and at the time of intensification of therapy (ΔCRP), coinciding with the biochemical mechanisms driving disease flare-ups. On the other hand, the main strength of the present study is that it compares two large and homogeneous cohorts of patients with IBD, with and without SARS-CoV-2 infection, reflecting real-world practice in the use of biological agents during the COVID-19 pandemic. In conclusion, the data reported in our study demonstrate that SARS-CoV-2 infection is not a significant risk factor for IBD recurrence in patients undergoing stable biological treatment. Furthermore, our results suggest that discontinuing biological treatment in patients with IBD after a diagnosis of COVID-19 is detrimental and should be avoided as much as possible. However, further studies are required to confirm these findings also in consideration of the spread of new variants of SARS-CoV-2, which could show pathogenetic characteristics that differ from those of the current strains.

## Figures and Tables

**Table 1:** Clinical characteristics of the study population.

diviVariable	All Patients (*n* = 285)	SARS-CoV-2 + ve Patients (*n* = 95)	Controls (*n* = 190)	*p*-Value
Age (years)	42 (30–55)	40 (30–55)	42 (32–44)	0.476
Sex (male)	158 (55.4%)	49 (51.6%)	109 (57.4%)	0.354
IBD diagnosis - CD	192 (67.4%)	64 (67.4%)	128 (67.4%)	0.724
- UC	93 (32.6%)	31 (32.6%)	62 (32.6%)	
Disease duration (years)	9 (5–15)	9 (5–15)	9 (5–16)	0.715
CD localization (L)				
- L1 - L2 - L3 - L4	95 (49.5%) 12 (6.2%) 83 (43.2%) 2 (1.1%)	31 (48.4%) 7 (10.9%) 26 (40.6%) 0	64 (50.0%) 5 (4.0%) 57 (44.4%) 2 (1.6%)	0.227
- Perianal involvement	37 (19.3%)	13 (20.3%)	24 (19.4%)	0.876
UC extension (E)				
- E1 - E2 - E3	8 (8.6%) 33 (35.5%) 52 (55.9%)	4 (12.9%) 11 (35.5%) 16 (51.6%)	4 (6.4%) 22 (35.5%) 36 (58.1%)	0.467
Previous surgery	102 (35.8%)	44 (46.3%)	58 (30.5%)	0.009
Active smoker	72 (25.3%)	29 (30.5%)	43 (22.6%)	0.148
Extraintestinal manifestations	62 (21.8%)	23 (24.2%)	39 (20.5%)	0.477
Biological therapeutic agents				
- Adalimumab - Infliximab - Ustekinumab - Vedolizumab	130 (45.6%) 90 (31.6%) 32 (11.2%) 33 (11.6%)	42 (44.2%) 30 (31.6%) 11 (11.6%) 12 (12.6%)	88 (46.3%) 60 (31.6%) 21 (11.1%) 21 (11.1%)	0.975
Steroids	9 (3.2%)	5 (5.3%)	4 (2.1%)	0.166
Mesalazine	59 (20.7%)	15 (15.8%)	44 (23.2%)	0.148
Immunosuppressants	9 (3.2%)	2 (2.1%)	7 (3.7%)	0.166
CRP level (mg/dL) (at the start of follow-up)	0.5 (0.5–2.2)	0.5 (0.5–3.2)	0.5 (0.5–1.7)	0.722
CRP level (mg/dL) (at the end of follow-up)	0.5 (0.5–2.6)	0.5 (0.5–2.8)	0.5 (0.5–2.0)	0.471
ΔCRP (mg/dL)	0 (0–0)	0 (0–0.2)	0 (0–0)	0.652

Values represent absolute numbers (%) or medians (interquartile range), and *p*-values < 0.05 were considered statistically significant. Abbreviations: SARS-CoV-2, severe acute respiratory syndrome coronavirus 2; +ve, positive; IBD, inflammatory bowel disease; CD, Crohn’s disease; UC, ulcerative colitis; CRP, C-reactive protein.

**Table 2:** Univariate analysis to determine factors associated with IBD relapse.

Variable	IBD Relapse *n* = 32	IBD in Remission *n* = 253	*p* Value
Age (years)	47 (32–66)	41 (30–54)	0.103
Sex (male)	17 (53.1%)	141 (55.7%)	0.780
IBD diagnosis			
- CD - UC	20 (62.5%) 12 (37.5%)	168 (66.4%) 85 (33.6%)	0.661
Disease duration (years)	10 (5–16)	9 (5–15)	0.935
Extraintestinal manifestations	8 (25.0%)	54 (21.3)	0.637
Previous surgery	11 (34.4%)	91 (36.0%)	0.859
Active smoker	11 (34.4%)	61 (24.1%)	0.208
Biological therapeutic agents			
- Adalimumab - Infliximab - Ustekinumab - Vedolizumab	14 (43.8%) 13 (40.6%) 3 (9.4%) 2 (6.3%)	116 (45.8%) 77 (30.4%) 29 (11.5%) 31 (12.3%)	0.582
Steroids	2 (6.3%)	7 (2.8%)	0.288
Mesalamine	9 (28.1%)	50 (19.8%)	0.271
Immunosuppressants	2 (6.3)	7 (2.8%)	0.267
CRP level (mg/dL) at the start of f-u	0.5 (0.5–4.5)	0.5 (0.5–2.1)	0.263
CRP level (mg/dL) at the end of f-u	0.5 (0.5–8.0)	0.5 (0.5–2.0)	0.086
ΔCRP (mg/dL)	0 (0–1.6)	0 (0–0)	0.002
Biological agent discontinuation	15 (46.9%)	38 (15.0%)	<0.001
SARS-CoV-2 infection	11 (34.4%)	84 (33.2%)	0.894
Follow-up duration (weeks)	20.3 (15.1–27.1)	22.3 (16.0–27.1)	0.751

Values represent absolute numbers (%) or medians (interquartile range), and *p*-values < 0.05 were considered statistically significant. Abbreviations: IBD, inflammatory bowel disease; CD, Crohn’s disease; UC, ulcerative colitis; CRP, C-reactive protein; SARS-CoV-2, severe acute respiratory syndrome coronavirus 2.

**Table 3:** Multivariate analysis to determine factors associated with IBD recurrence.

Variable	Multivariate *p*-Value	Hazard Ratio (95% Confidence Interval)
SARS-CoV-2 infection *	0.298	0.42 (0.08–2.15)
ΔCRP	0.017	1.14 (1.02–1.27)
Biological agent discontinuation	0.033	7.27 (1.17–45.18)

*p*-values < 0.05 were considered statistically significant. Abbreviations: IBD, inflammatory bowel disease; SARS-CoV-2, severe acute respiratory syndrome coronavirus 2; CRP, C-reactive protein. * SARS-CoV-2 diagnosis was forced in the analysis.

**Table 4:** Clinical characteristics of the 95 SARS-CoV-2-positive patients.

Variable	Crohn’s Disease (*n* = 64)	Ulcerative Colitis (*n* = 31)	*p*-Value
COVID-19 severity *			
- Asymptomatic - Mild - Severe	22 (34.4%) 41 (64.1%) 1 (1.6%)	11 (35.5%) 17 (54.8%) 3 (9.7%)	0.168
Sex (male)	33 (51.6%)	16 (51.6%)	1.000
Age (years)	39 (27–53)	43 (34–58)	0.090
Disease duration (years)	9 (5–15)	8 (4–17)	0.605
Extraintestinal manifestations	8 (25.0%)	54 (21.3%)	0.637
Previous surgery	44 (68.8%)	0	<0.001
Active smoker	21 (32.8%)	8 (25.8%)	0.487
Biological therapeutic agents			
- Adalimumab - Infliximab - Ustekinumab - Vedolizumab	34 (53.1%) 15 (23.4%) 10 (15.6%) 5 (7.8%)	8 (25.8%) 15 (48.4%) 1 (3.2%) 7 (22.6%)	0.003
Steroids	3 (4.7%)	2 (6.5%)	0.718
Mesalamine	1 (1.6%)	14 (45.2%)	<0.001
Immunosuppressants	2 (3.1%)	0	1.000
Extraintestinal manifestations	17 (26.6%)	6 (19.4%)	0.442
CRP level (mg/dL) at the start of f-u	0.5 (0.5–2.7)	0.5 (0.5–3.7)	0.336
CRP level (mg/dL) at the end of f-u	0.5 (0.5–3.1)	0.7 (0.5–2.8)	0.500
ΔCRP (mg/dL)	0.0 (0.2–0.0)	0.0 (0.5–1.2)	0.107
Discontinuation of treatment with biological agents	29 (45.3%)	17 (54.8%)	0.384
Discontinued doses	2 (2–2)	2 (1–2)	0.003
F-u duration (weeks)	20.3 (15.8–26.0)	23.3 (18.0–27.4)	0.304
Disease recurrence	6 (9.4%)	5 (16.1%)	0.335

Values represent absolute numbers (%) or medians (interquartile range), and *p*-values < 0.05 were considered statistically significant. Abbreviations: SARS-CoV-2, severe acute respiratory syndrome coronavirus 2; COVID-19, coronavirus disease 2019; CRP, C-reactive protein; f-u, follow-up. * COVID-19 severity was categorized as asymptomatic (no symptoms), mild (requiring no oxygen supplementation or hospitalization), and severe (requiring hospitalization or oxygen supplementation).

## Data Availability

The data presented in this study are available on request from the corresponding author. The data are not publicly available due to privacy restrictions.
